# Supplementary material for: Multi‐dataset identification of innovative feature genes and molecular mechanisms in keratoconus
Source: J Cell Mol Med. 2024 Sep 19;28(18):e70079. doi: 10.1111/jcmm.70079 (PMC11412914; doi:10.1111/jcmm.70079)
Supplement: Supplementary file 2 — Material S2. [file JCMM-28-e70079-s002.zip › 7.GSEA analysis/1.ARL11/KEGG_TYROSINE_METABOLISM.html]

Details for gene set KEGG\_TYROSINE\_METABOLISM[GSEA]

|  || Dataset | ARL11.group.cls#h\_versus\_l |
| Phenotype | group.cls#h\_versus\_l |
| Upregulated in class | l |
| GeneSet | KEGG\_TYROSINE\_METABOLISM |
| Enrichment Score (ES) | -0.31858534 |
| Normalized Enrichment Score (NES) | -1.0363067 |
| Nominal p-value | 0.42454728 |
| FDR q-value | 1.0 |
| FWER p-Value | 0.999 |
Table: GSEA Results Summary

  

Fig 1: Enrichment plot: KEGG\_TYROSINE\_METABOLISM      
 Profile of the Running ES Score & Positions of GeneSet Members on the Rank Ordered List

  

| SYMBOL | TITLE | RANK IN GENE LIST | RANK METRIC SCORE | RUNNING ES | CORE ENRICHMENT || 1 | MAOB | na | 1064 | 0.382 | 0.0502 | No |
| 2 | AOC2 | na | 1449 | 0.342 | 0.1114 | No |
| 3 | ADH4 | na | 3617 | 0.215 | 0.0943 | No |
| 4 | IL4I1 | na | 4200 | 0.194 | 0.1184 | No |
| 5 | PNMT | na | 5429 | 0.154 | 0.1155 | No |
| 6 | ADH6 | na | 6053 | 0.136 | 0.1262 | No |
| 7 | TRMT11 | na | 6943 | 0.113 | 0.1245 | No |
| 8 | TH | na | 8527 | 0.082 | 0.0963 | No |
| 9 | AOX1 | na | 8738 | 0.079 | 0.1069 | No |
| 10 | GOT2 | na | 9613 | 0.066 | 0.0956 | No |
| 11 | METTL2B | na | 10273 | 0.057 | 0.0886 | No |
| 12 | LCMT2 | na | 10722 | 0.051 | 0.0864 | No |
| 13 | ALDH3B1 | na | 11840 | 0.038 | 0.0622 | No |
| 14 | MAOA | na | 12281 | 0.034 | 0.0566 | No |
| 15 | ALDH3B2 | na | 13954 | 0.019 | 0.0125 | No |
| 16 | FAH | na | 16009 | 0.004 | -0.0460 | No |
| 17 | ADH1B | na | 16426 | 0.001 | -0.0578 | No |
| 18 | COMT | na | 16431 | 0.001 | -0.0578 | No |
| 19 | DCT | na | 17406 | -0.006 | -0.0846 | No |
| 20 | MIF | na | 18538 | -0.014 | -0.1142 | No |
| 21 | ADH7 | na | 18569 | -0.014 | -0.1121 | No |
| 22 | TYRP1 | na | 18888 | -0.016 | -0.1178 | No |
| 23 | GSTZ1 | na | 19033 | -0.017 | -0.1184 | No |
| 24 | LCMT1 | na | 19341 | -0.019 | -0.1231 | No |
| 25 | GOT1 | na | 19508 | -0.021 | -0.1236 | No |
| 26 | ALDH3A1 | na | 20262 | -0.026 | -0.1398 | No |
| 27 | DDC | na | 23583 | -0.057 | -0.2235 | No |
| 28 | TPO | na | 23798 | -0.060 | -0.2171 | No |
| 29 | ADH5 | na | 23842 | -0.060 | -0.2056 | No |
| 30 | TAT | na | 24898 | -0.075 | -0.2202 | No |
| 31 | TYR | na | 26252 | -0.094 | -0.2393 | No |
| 32 | METTL6 | na | 26386 | -0.096 | -0.2227 | No |
| 33 | HPD | na | 27499 | -0.119 | -0.2297 | No |
| 34 | HEMK1 | na | 28577 | -0.145 | -0.2300 | No |
| 35 | ALDH1A3 | na | 28596 | -0.146 | -0.1996 | No |
| 36 | AOC3 | na | 32726 | -0.302 | -0.2547 | Yes |
| 37 | HGD | na | 33078 | -0.327 | -0.1956 | Yes |
| 38 | ADH1C | na | 33242 | -0.340 | -0.1284 | Yes |
| 39 | ADH1A | na | 33507 | -0.360 | -0.0599 | Yes |
| 40 | DBH | na | 34180 | -0.450 | 0.0158 | Yes |
Table: GSEA details [plain text format]

  

Fig 2: KEGG\_TYROSINE\_METABOLISM      
 Blue-Pink O' Gram in the Space of the Analyzed GeneSet

  

Fig 3: KEGG\_TYROSINE\_METABOLISM: Random ES distribution      
 Gene set null distribution of ES for **KEGG\_TYROSINE\_METABOLISM**
